# Untreated Isolated Sytolic Hypertension among Middle-Aged and Old Adults in the United States: Trends in the Prevalence by Demographic Factors During 1999–2010

**DOI:** 10.1155/2015/508584

**Published:** 2015-02-23

**Authors:** Xuefeng Liu, Van Minh Hoang, Yali Liu, Rachel L. W. Brown

**Affiliations:** ^1^Department of Systems Leadership and Effective Science, University of Michigan, Ann Arbor, MI 48109, USA; ^2^Institute for Quantitative Biology, East Tennessee State University, Johnson City, TN 37614, USA; ^3^Department of Health Economics and Center for Health System Research, Hanoi Medical University, Hanoi, Vietnam; ^4^Institute for Preventive Medicine and Public Health, Hanoi Medical University, Hanoi, Vietnam; ^5^Department of Mathematics and Statistics, East Tennessee State University, Johnson City, TN 37614, USA; ^6^Department of Pharmaceutical Practice, East Tennessee State University, Johnson City, TN 37614, USA

## Abstract

Isolated systolic hypertension (ISH) predominates hemodynamic hypertension subtypes and becomes a significant factor for cardiovascular and renal outcomes in middle-aged and old adults. The prevalence and changes of untreated ISH have not been fully investigated in this population. A total of 12,097 participants aged ≥40 years were selected from the National Health and Nutrition Examination Survey 1999–2010. The overall prevalence of untreated ISH was 15.2%. The prevalence decreased significantly from 16.8% in 1999–2004 to 13.5% in 2005–2010. Females, non-Hispanic blacks, and adults with low education had higher prevalence of untreated ISH than males, non-Hispanic whites, and adults with high education, respectively. Compared with 1999–2004, the prevalence of untreated ISH in 2005–2010 reduced in old adults (28.0% versus 37.7%), females (14.3% versus 19.5%), and non-Hispanic whites (12.7% versus 16.2%). The stratified prevalence of untreated ISH decreased in 2005–2010 in non-Hispanic white females (12.8% versus 18.6%) and females who did not attend college (16.9% versus 21.8%). Untreated ISH is more prevalent in old and female subjects, and significant improvements in these groups suggest that public health measures or changes are in the right direction.

## 1. Introduction

Hypertension includes three hemodynamic subtypes: isolated systolic hypertension (ISH), systolic-diastolic hypertension, and isolated diastolic hypertension. ISH has been defined as a systolic blood pressure (SBP) of at least 140 mm Hg and a diastolic blood pressure (DBP) of less than 90 mm Hg [1]. Typically, ISH predominates as the major form of hypertension for older people after the age of 50 years as SBP continues to rise and DBP tends to fall [2]. It is often characterized as a phenomenon of aging [3, 4]. Risk factors for ISH include being older, being overweight, smoking, and having diabetes. The main cause of ISH may be related to age-associated changes in the blood vessels [5]. If ISH is left uncontrolled, it can lead to heart attack, stroke, and incident heart failure [6–8].

ISH is a growing concern in older adults, and a large percentage of adults are not appropriately managed [9]. Although the prevalence of ISH has been reported in several international studies [10–12], inconsistent age selections between studies limit available results on ISH prevalence in middle-aged and old adults. Several existing studies of ISH in the US adult population focus on the percentage distribution of hypertension subtypes with age among uncontrolled hypertensive subjects, and the prevalence of untreated ISH in the general population of middle-aged and old people has not been investigated [13, 14]. In addition, the studies are based on the third National Health and Nutrition Examination Survey (NHANES III, 1988–1994) and cannot reflect changes in the prevalence or distribution of related risk factors (e.g., obesity) that occurred during the last decade. Another recent study of ISH examines the prevalence of ISH in the US black population and has not reported the prevalence in the general middle-aged or old population [15].

In this study, sociodemographic factors (including age, gender, race, and education) and blood pressure (BP) measurements were collected from NHANES 1999–2010 and then analyzed to estimate the prevalence of untreated ISH in middle-aged and old people in the Unites States (US). We followed the advisory guidelines from the National High Blood Pressure Education Program with the expressed purpose of further characterizing systolic hypertension. Specific attention was directed toward estimating the prevalence of untreated ISH in the population of adults aged 40 years or older and in the subpopulations of these adults stratified by demographic factors (age, gender, race/ethnicity, and education). As the population in the US is aging and ISH is more prevalent in middle-aged and old people, estimating the prevalence of untreated ISH, particularly the stratified prevalence in different subpopulation, would be critical for identifying the distribution of untreated ISH among people with differential demographic background and reducing the risks of adverse cardiovascular and renal events in adults at higher hypertension risk.

## 2. Methods

### 2.1. Study Design and Population

The NHANES program was conducted by the National Center for Health Statistics, the US Centers for Disease Control and Prevention (CDC). Beginning in 1999, the continuous NHANES included a series of two-year cross sectional surveys designed to assess the health and nutrition status among children and adults in the US [16]. In each cycle of the survey, a nationally representative sample of the US civilian noninstitutionalized population was selected across the country through a stratified multistage probability clustered sampling design. All surveys consisted of interviews and examination. Interviews were performed in the participants' home to obtain information regarding sociodemographic characteristics and history of diseases. Examination was conducted in the Mobile Examination Center to obtain measurements about examination and clinical/laboratory factors. All adults provided written informed consent and the data were approved by the CDC Institutional/Ehtics Review Board to ensure confidentiality.

Demographic and examinational data used in the present study were collected from the six cycles of NHANES from 1999 to 2010 to increase sample sizes and improve power for subgroup analyses. The sample from each survey was weighted to the US population corresponding to the respective time periods during which the surveys were performed. The stratified multistage probability clustered sampling design was similar from NHANES 1999-2000 to NHANES 2009-2010. We compared the 6-year prevalence of untreated ISH between 1999–2004 and 2005–2010. For the prevalence estimate of untreated ISH in NHANES 1999–2004, a 6-year weight was created by assigning two thirds of the 4-year weight for NHANES 1999–2002 and one third of the 2-year weight for NHANES 2003-2004; for the prevalence estimate in NHANES 2005–2010, a 6-year weight was created by assigning one third of the 2-year weight for NHANES 2005-2006, 2007-2008, and 2009-2010, respectively.

Since the goal of this study was to estimate the prevalence and changes of untreated ISH in middle-aged and old adults by demographic factors, the participants aged <40 years were excluded from the study. We also excluded those persons who only participated in the interview or examination but not in both, who had incomplete SBP or DBP measurements, or who were taking antihypertensive medications for hypertension when being interviewed. Finally, a total of 12,097 participants were included in the present study, among which 5,757 persons were from NHANES 1999–2004 and 6,340 persons from NHANES 2005–2010.

### 2.2. Isolated Systolic Hypertension Assessment

Blood pressure in NHANES 1999–2010 was measured by trained physicians in a standardized procedure using mercury sphygmomanometer and appropriate-size arm cuffs [17]. Participants were recorded up to four SBP and DBP readings after resting for 5 minutes quietly in a sitting position. The average SBP and DBP were calculated based on individual readings according to the NHANES specifications. A participant was determined to have isolated systolic hypertension if his/her average SBP was ≥140 mm Hg and average DBP was <90 mm Hg.

### 2.3. Demographic Factors of Interest

Four characteristics (age, gender, race, and education) were selected to see differences in the prevalence of untreated ISH across the factors. Sociodemographic information on age, gender, race, and education was assessed through questionnaire files of NHANES. Participants were divided into two groups in terms of age: middle-aged adults if ages are greater than or equal to 40 years and less than 65 years and old adults if ages are 65 years or above. The survey population includes four races of people: Hispanics (including non-Mexican Hispanics and Mexican Americans), non-Hispanic whites, non-Hispanic blacks, and other races. The participant's level of education was classified as receiving education of high school or below and college or above, based on the number of years in school.

### 2.4. Statistical Analysis

NHANES Analytic and Reporting Guidelines were followed [18]. The study sample with a stratified multistage sampling design was obtained by combining six two-year cycles of continuous NHANES data to estimate overall prevalence of untreated ISH during 1999–2010. The 6-year prevalence of untreated ISH was estimated to examine differences/changes in the prevalence between 1999–2004 and 2005–2010. To reflect unequal probabilities of selection, nonresponse adjustments and poststratification adjustments, examination sampling weights were incorporated into the data analysis to get bias-reduced estimates and sampling errors of estimates. Sampling weights were also used to adjust for the impact of oversampling non-Hispanic blacks, Hispanics, and individuals aged 60 years or older in the NHANES survey [18].

Since continuous NHANES was a national survey with a multistage clustered probability sampling design, conventional statistical analyses with underlying distributional assumptions were not appropriate for variance estimation and statistical testing [18]. Survey procedures were used to compute Taylor series standard errors, independent *t*-tests were used to compare means of continuous characteristics and *χ* ^2^, and the Cochran-Mantel-Haenszel tests were used for categorical survey data. We calculated age-adjusted descriptive statistics for subject characteristics using PROC SURVEYMEANS for continuous variables and PROC SURVEYREG for categorical values. The age-adjusted prevalence and 95% confidence intervals (CIs) of untreated ISH were estimated by conducting weighted simple linear models. Odds ratios (ORs) and 95% CIs of untreated ISH prevalence were calculated from weighted multiple logistic regression models to examine the impact of each demographic factor on the prevalence of untreated ISH after controlling for others. Statistical analyses were performed on PC with Microsoft Window 7 operating system, using SAS statistical software (SAS version 9.2, SAS Institute Inc., Cary, NC, USA).

## 3. Results

Average age of adults in the present study was 54.3 years. 51.3% were women, 10.0% were Hispanics, 77.7% were non-Hispanic whites, and 7.8% were non-Hispanic blacks. 45.1% of adults received high school education or below. There were no significant differences in the related characteristics between NHANES 1999–2004 and NHANES 2005–2010 (Table 1).

**Figure 1:**
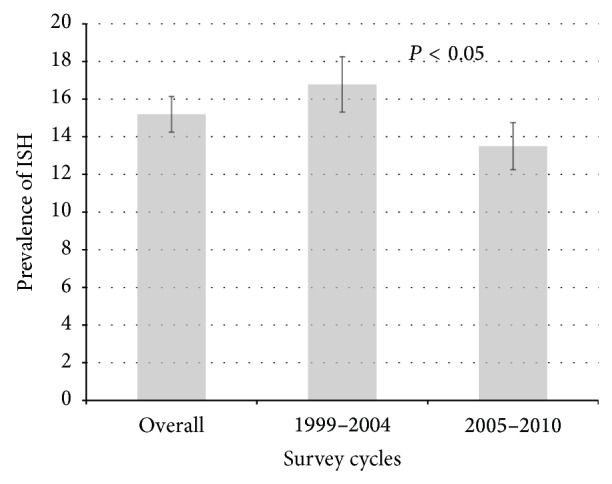
Prevalence and 95% confidence intervals of isolated systolic hypertension by survey cycles in NHANES 1999–2010, USA. (The prevalence of isolated systolic hypertension was age-adjusted by direct standardization to the 2000 projected US population. NHANES, National Health and Nutrition Examination Survey; ISH, isolated systolic hypertension. *P* < 0.05 indicates the significant difference in the prevalence of ISH between 1999–2004 and 2005–2010).

The prevalence of untreated ISH in middle-aged and old adults is shown in Figure 1. The overall prevalence of untreated ISH during 1999–2010 is 15.2% (95% CI: 14.2–16.1%). The 6-year prevalence of untreated ISH was 16.8% in 1999–2004 and 13.5% in 2005–2010. The prevalence decreased significantly from 1999–2004 to 2005–2010 (*P* < 0.05).

Table 2 presents the prevalence and changes of untreated ISH by the 4 demographic factors in middle-aged and old adults in NHANES 1999–2010. The prevalence of untreated ISH was higher in adults aged ≥65 years than in adults aged 40–64 years. Females had higher prevalence of untreated ISH (16.9%, 95% CI: 15.8–18.1%) than males (13.3%, 95% CI: 12.1–14.4%). Non-Hispanic blacks had higher prevalence of untreated ISH (20.4%, 95% CI: 18.4–22.3%) than non-Hispanic whites (14.5%, 95% CI: 13.4–15.6%). Individuals with high school education or below had higher prevalence (17.6%, 95% CI: 16.3–18.8%) than those with college education or above (13.1%, 95% CI: 11.8–14.4%). Compared with 1999–2004, the prevalence of untreated ISH in 2005–2010 decreased significantly in old (28.0%, 95% CI: 25.1–30.9% versus 37.7%, 95% CI: 34.8–40.6%), female (14.3%, 95% CI: 12.7–15.8% versus 19.5%, 95% CI: 17.8–21.1%), and non-Hispanic white (12.7%, 95% CI: 11.3–14.2% versus 16.2%, 95% CI: 14.6–17.9%) individuals.

Prevalence estimates and changes of untreated ISH stratified by gender and race, gender and education, and education and race are presented in Table 3. From this table, we can see that the stratified prevalence of untreated ISH in 2005–2010 was not shown to be significantly different from that in 1999–2004 except for non-Hispanic white females (12.8%, 95% CI: 11.1–14.5% in 2005–2010 versus 18.6%, 95% CI: 16.7–20.6% in 1999–2004) and females who did not attend college (16.9%, 95% CI: 14.6–19.2% in 2005–2010 versus 21.8%, 95% CI: 19.2–24.3% in 1999–2004). Non-Hispanic black males had higher prevalence of untreated ISH than other racial males (black males: 18.0%, 95% CI: 16.0–19.9% versus white males: 13.0%, 95% CI: 11.7–14.4%; Hispanic males: 12.9%, 95% CI: 10.5–15.3%; and other males: 9.2%, 95% CI: 4.9–13.4%), and non-Hispanic black females had higher prevalence than non-Hispanic white females (22.7%, 95% CI: 19.2–26.3% versus 15.8%, 95% CI: 14.5–17.0%). Individuals with high school education or below had higher prevalence of untreated ISH than individuals with college education or above in both males (15.1%, 95% CI: 13.6–16.6% versus 11.8%, 95% CI: 10.4–13.2%) and females (19.6%, 95% CI: 17.9–21.3% versus 14.4%, 95% CI: 12.5–16.2%). Stratification analysis by education and race indicated that among individuals with high school education or below, the prevalence of untreated ISH was higher in non-Hispanic blacks (22.9%, 95% CI: 20.4–25.4%) than in non-Hispanic whites (16.9%, 95% CI: 15.3–18.4%).

Table 4 shows odds ratios and 95% confidence intervals of untreated ISH by demographic factors among middle-aged and old adults in NHANES 1999–2010. Untreated ISH was more likely to be prevalent in older adults, non-Hispanic blacks, and adults who received a high school education or below in either period of 1999–2004 or 2005–2010. Compared with males, females had higher chance to have untreated ISH in 1999–2004 but not in 2005–2010.

## 4. Discussion

The NHANES data have been used to study the prevalence of hypertension in the United States [19, 20], racial disparity in cardiovascular risk factors [21], and the pattern of hypertension subtypes [13, 14]. We used the NHANES 1999–2010 to estimate the prevalence and changes of untreated ISH in the population of middle-aged and old US adults by age, gender, race/ethnicity, and education. The present study demonstrated that the prevalence of untreated ISH in middle-aged and old adults decreased significantly from 16.8% in 1999–2004 to 13.5% in 2005–2010. The decrease in the prevalence of untreated ISH in old adults (37.7% versus 28.0%), females (19.5% versus 14.3%), and non-Hispanic whites (16.2% versus 12.7%) could explain this change. Females, non-Hispanic blacks, and low-educated individuals had higher prevalence of untreated ISH than males, non-Hispanic whites, and individuals with higher education, respectively. Stratified analyses showed that the decrease in the prevalence of untreated ISH in females from 1999–2004 to 2005–2010 might be contributed by the significant decrease in the prevalence in non-Hispanic white females and females with lower education and that the higher prevalence of untreated ISH in non-Hispanic blacks might be contributed by the prevalence in non-Hispanic black males higher than other racial males and the prevalence in non-Hispanic black females higher than non-Hispanic white females. Further analyses indicated that individuals with lower education had higher prevalence of untreated ISH than individuals with higher education in both males and females, and the prevalence of untreated ISH was higher in poorly educated non-Hispanic blacks.

The decrease in the prevalence of untreated ISH in the overall population of adults aged ≥40 years and the population of old adults coincided with increasing trend of hypertensive patients on treatment, particularly in old people [20]. A greater percentage of hypertensive patients receiving treatment suggest that more patients with isolated systolic hypertension, particularly in old individuals, are receiving antihypertensive medications to lower high BP since ISH is a major form of hypertension subtypes in old people. Over the 12-year period from 1999 through 2010, obesity did not show the significant decrease in women and non-Hispanic whites [22]. Healthy lifestyles are an unlikely explanation for the decrease in untreated ISH prevalence in females and non-Hispanic whites from 1999–2004 to 2005–2010, because eating patterns became less “DASH-like” [23] and obesity did not decrease over time [22, 24].

Compared with non-Hispanic whites, the higher prevalence of untreated ISH in non-Hispanic blacks is consistent with the higher prevalence of hypertension from the previous study [19, 20]. Obesity has been shown to be an important determinant of untreated ISH [24]. Non-Hispanic blacks have significantly higher prevalence of obesity than in non-Hispanic whites [21, 22]. In addition, non-Hispanic blacks are poorer and receive less education than non-Hispanic whites [21]; poverty and low levels of education are associated with the increased risk of overweight or obesity [25]. The higher prevalence of untreated ISH in blacks than in whites and the higher prevalence in individuals with lower education may be attributed to the racial/ethnic differences in the prevalence of obesity.

The US population is aging and ISH is the most common hypertension subtype in middle-aged and old adults, particularly in old adults. The decreased prevalence of untreated ISH in old adults in this study may have significant clinic indications. Every 20 mm Hg increase in SBP is associated with doubling of mortality from both coronary heart disease and stroke in people between the ages of 40 and 89 years [26]. The absolute risk of CV mortality for any given increase in SBP is much more pronounced in the older population [27]. The significant decrease in the prevalence of untreated ISH may lower the risks of coronary heart disease and stroke and extend the overall life expectancy and life expectancy free of CV disease in old adults. The reason for the lowered prevalence in recent 6 years compared with 1999–2004 may be due to the increased awareness of hypertension, greater concerns in health status, and improved lifestyles in older people [19, 28].

Non-Hispanic whites account for the majority of hypertensive population in the US [19, 20]. The significant decrease in the prevalence of untreated ISH in non-Hispanic whites may contribute to the significant decrease in the number of hypertensive patients and reduce ISH-related cardiovascular and renal outcomes in middle-aged and old population. SBP has been shown to be a stronger risk factor of myocardial infarction for women compared with men [29]. Among women aged 65–84 years, the hazard of death significantly increased with increase in SBP [30]. In this study, the decreased prevalence of untreated ISH in females, particularly in old females in 2005–2010, may reduce the risk of myocardial infarction and CV-related mortality in old women in the future. The cause of the decreased prevalence of untreated ISH in females remains unclear. The possible reasons may include the greater awareness of hypertension and the lack of increase in body mass index in females, particularly in old females [20, 23].

One of the national goals of Healthy People 2020 concerning hypertension is that the proportion of adults with hypertension should be reduced to 26.9% [30]. It seems unlikely to attain this goal because of the aging population. However, the decreased prevalence of untreated ISH in old adults, females, and non-Hispanic whites in the last few years is promising, suggesting that the target of 26.9% may be reached by 2020 with concerted efforts from health professionals and government agencies. Overall decreased trends in the prevalence of untreated ISH in the subpopulations stratified by combinations of gender, race, and education are promising although some are not statistically significant and are still in need of substantial improvement. The prevalence of untreated ISH is relatively high and does not decline from 1999–2004 to 2005–2010 in non-Hispanic females, Hispanic females, and non-Hispanic blacks with education of high school or below. To meet Healthy People 2020, the greater attention must be paid to these groups. Therefore, there is room for further improvement in the control of hypertension in the United States.

There are several limitations in our study. Hypertensive individuals who took prescribed medications for treatment were not included in the study because we could not determine what types of hypertension they had (isolated systolic hypertension, isolated diastolic hypertension, or systolic and diastolic hypertension) due to antihypertensive drug therapy. Exclusion of hypertensive adults under antihypertensive treatment may underestimate the prevalence of ISH in the general US population. The prevalence estimated in the present study is the prevalence of untreated ISH in USA. A few individuals (*n* = 342) who were diagnosed to have hypertension used nonpharmacological techniques to control hypertension. The information for hypertension subtypes in the hypertensive persons with SBP successfully controlled by physical activity, weight control, and other nonpharmacological techniques was not available. Our definition of ISH treated them as normotensive persons, and this might also underestimate the prevalence of untreated ISH in the study. The increasing rate of Type I error was another concern by using independent *t*-tests when multiple comparisons of the prevalence of untreated ISH occurred across the multiply stratified factors.

## 5. Perspectives

ISH predominates and becomes a major form of hypertension subtypes in middle-aged and old adults. Our study indicated that there were no significant changes in the prevalence of untreated ISH in middle-aged individuals and in males from 1999–2004 to 2005–2010. However, the findings that untreated ISH was more prevalent in old adults and in females and the prevalence of untreated ISH improved significantly for these two subpopulations in recent years suggest that public health measures or changes in clinical practice are in the right direction. The higher prevalence of untreated ISH in non-Hispanic blacks compared with other races and no significant improvements reveal that more attention should be paid to this racial population in the future.

## Figures and Tables

**Table 1:** Characteristics of participants in NHANES 1999–2010, USA.

Characteristics	Mean or percentage (95% CI)		
	Overall	NHANES 1999–2004	NHANES 2005–2010
Age (years)	54.3 (53.9, 54.6)	54.4 (54.0, 54.8)	54.2 (53.7, 54.7)
Gender			
Female, %	51.3 (50.3, 52.2)	51.2 (49.7, 52.7)	51.3 (50.1, 52.5)
Race			
Hispanic, %	10.0 (8.2, 11.8)	9.7 (6.8, 12.7)	10.2 (8.1, 12.4)
Non-Hispanic white, %	77.7 (75.5, 79.9)	78.7 (75.4, 81.9)	76.7 (73.5, 79.9)
Non-Hispanic black, %	7.8 (6.7, 8.8)	7.6 (6.1, 9.1)	7.9 (6.5, 9.4)
Other races, %	4.5 (3.9, 5.3)	4.0 (3.0, 4.9)	5.2 (4.1, 6.3)
Education			
High school or below, %	45.1 (43.3, 46.9)	46.8 (44.1, 49.4)	43.5 (40.9, 46.1)

Note: data were age-adjusted (except for age-specific estimates) by direct standardization to the 2000 projected US population.

NHANES: National Health and Nutrition Examination Survey; CI: confidence interval.

**Table 2:** Prevalence and changes of untreated isolated systolic hypertension by age, gender, race, and education in NHANES 1999–2010, USA.

Characteristics	Prevalence (95% CI)		
	Overall, %	NHANES 1999–2004, %	NHANES 2005–2010, %
Age (years)			
40–64	15.6 (13.5, 17.7)	16.2 (13.0, 19.6)	15.1 (12.5, 17.6)
≥65	33.1 (31.1, 35.1)	37.7 (34.8, 40.6)	28.0 (25.1, 30.9)^*^
Gender			
Male	13.3 (12.1, 14.4)	13.8 (12.1, 15.5)	12.6 (11.1, 14.1)
Female	16.9 (15.8, 18.1)	19.5 (17.8, 21.1)	14.3 (12.7, 15.8)^*^
Race			
Hispanic	17.3 (14.6, 19.9)	18.3 (14.0, 22.7)	16.0 (13.4, 18.5)
Non-Hispanic white	14.5 (13.4, 15.6)	16.2 (14.6, 17.9)	12.7 (11.3, 14.2)^*^
Non-Hispanic black	20.4 (18.4, 22.3)	21.7 (19.2, 24.2)	18.9 (15.8, 22.0)
Other races	14.0 (9.9, 18.0)	15.7 (8.1, 23.3)	12.6 (8.2, 17.0)
Education			
High school or below	17.6 (16.3, 18.8)	18.8 (16.7, 20.9)	16.0 (14.5, 17.5)
College or above	13.1 (11.8, 14.4)	14.9 (13.2, 16.7)	11.4 (9.6, 13.3)

Note: the prevalence of isolated systolic hypertension was age-adjusted (except for age-specific estimates) by direct standardization to the 2000 projected US population.

NHANES: National Health and Nutrition Examination Survey; CI: confidence interval.

^*^indicates that the difference in the prevalence of untreated ISH between 1999–2004 and 2005–2010 is significant at the 0.05 level.

**Table 3:** Prevalence and changes of untreated isolated systolic hypertension stratified by characteristics in NHANES 1999–2010, USA.

Characteristics	Stratified prevalence (95% CI)		
	Overall, %	NHANES 1999–2004, %	NHANES 2005–2010, %
Stratified by gender and race			
Male			
Hispanic	12.9 (10.5, 15.3)	12.8 (8.7, 17.0)	12.9 (10.3, 15.5)
Non-Hispanic white	13.0 (11.7, 14.4)	13.6 (11.5, 15.6)	12.4 (10.6, 14.3)
Non-Hispanic black	18.0 (16.0, 19.9)	20.6 (18.3, 23.0)	15.7 (12.7, 18.8)
Other races	9.2 (4.9, 13.4)	8.5 (1.2, 15.8)	9.5 (4.2, 14.8)
Female			
Hispanic	21.6 (17.8, 25.3)	23.4 (17.9, 28.9)	19.1 (15.0, 23.3)
Non-Hispanic white	15.8 (14.5, 17.0)	18.6 (16.7, 20.6)	12.8 (11.1, 14.5)^*^
Non-Hispanic black	22.7 (19.2, 26.3)	22.7 (18.5, 26.9)	22.4 (16.0, 28.7)
Other races	18.5 (12.2, 24.7)	20.8 (10.6, 31.0)	15.8 (8.3, 23.3)

Stratified by gender and education			
Male			
High school or below	15.1 (13.6, 16.6)	15.2 (12.7, 17.7)	14.9 (13.1, 16.7)
College or above	11.8 (10.4, 13.2)	12.7 (10.8, 14.6)	10.9 (8.9, 13.0)
Female			
High school or below	19.6 (17.9, 21.3)	21.8 (19.2, 24.3)	16.9 (14.7, 19.2)^*^
College or above	14.4 (12.5, 16.2)	17.2 (14.4, 20.0)	11.9 (9.5, 14.3)

Stratified by education and race			
High school or below			
Hispanic	18.6 (16.0, 21.2)	19.4 (15.2, 23.6)	17.5 (14.7, 20.3)
Non-Hispanic white	16.9 (15.3, 18.4)	18.4 (15.9, 21.0)	15.0 (13.2, 16.8)
Non-Hispanic black	22.9 (20.4, 25.4)	23.4 (20.8, 26.1)	21.8 (17.5, 26.1)
Other races	15.7 (9.6, 21.8)	15.4 (6.2, 24.6)	15.6 (8.0, 23.3)
College or above			
Hispanic	12.9 (8.2, 17.6)	15.0 (7.9, 22.1)	11.3 (5.7, 17.0)
Non-Hispanic white	12.8 (11.5, 14.2)	14.7 (12.9, 16.5)	11.1 (9.1, 13.2)
Non-Hispanic black	16.0 (12.8, 19.3)	18.1 (12.0, 24.2)	14.8 (11.0, 18.5)
Other races	12.6 (8.0, 17.2)	15.1 (5.0, 25.2)	11.2 (6.4, 15.9)

The stratified prevalence of isolated systolic hypertension was age-adjusted by direct standardization to the 2000 projected US population.

NHANES: National Health and Nutrition Examination Survey; CI: confidence interval.

^*^indicates that the stratified difference in the prevalence of untreated ISH between 1999–2004 and 2005–2010 is significant at the 0.05 level.

**Table 4:** Odds ratios and 95% confidence intervals of isolated systolic hypertension by demographic factors in NHANES 1999–2010, USA.

Factors	Odds ratios (95% CI)	
	NHANES 1999–2004	NHANES 2005–2010
Age (reference: 40–49 years)		
50–64 years	3.6 (2.7, 5.0)^‡^	3.3 (2.5, 4.5)^‡^
≥65 years	14.8 (10.5, 20.8)^‡^	9.90 (7.3, 13.4)^‡^
Gender (reference: male)		
Female	1.5 (1.3, 1.8)^‡^	1.1 (0.9, 1.30)
Race/ethnicity (reference: non-Hispanic white)		
Hispanic	1.0 (0.7, 1.5)	1.2 (0.9, 1.5)
Non-Hispanic black	1.6 (1.3, 2.0)^†^	1.8 (1.4, 2.3)^‡^
Other races	0.90 (0.5, 1.7)	1.1 (0.7, 1.7)
Education (reference: college or above)		
High school or below	1.4 (1.1, 1.7)^†^	1.5 (1.2, 1.9)^†^

NHANES: National Health and Nutrition Examination Survey; CI: confidence interval.

^‡^ *P* < 0.0001 and ^†^ *P* < 0.01 for the odds ratio of isolated systolic hypertension for each factor relative to the corresponding reference category.
